# Correction: Catalytic conversion of carbon dioxide (CO_2_) using coal-based nano-carbon materials

**DOI:** 10.1039/d4ra90103g

**Published:** 2024-09-20

**Authors:** Hongchao Luo, Xinjuan Liu

**Affiliations:** a School of Chemistry and Materials Engineering, Liupanshui Normal University Guizhou Province China luohongchao@lpssy.edu.cn; b School of Environmental and Chemical Engineering, Dalian University Dalian 116622 Liaoning Province China

## Abstract

Correction for ‘Catalytic conversion of carbon dioxide (CO_2_) using coal-based nano-carbon materials’ by Hongchao Luo *et al.*, *RSC Adv.*, 2024, **14**, 27298–27309, https://doi.org/10.1039/D4RA03407D.

The authors regret that incorrect details were given for ref. 16 in the original article. The correct version of ref. 16 is given below as ref. 1.

In addition, the author contributions were incorrectly given. The corrected contributions are as shown here.

**Author contributions:**

Hongchao Luo: writing – review & editing, proof checking, conceptualization, supervision, project administration and funding acquisition. Xinjuan Liu: data curation, writing – original draft.

The Royal Society of Chemistry apologises for these errors and any consequent inconvenience to authors and readers.
